# Analysis of Three Potential Savings in E-Working Expenditure

**DOI:** 10.3389/fsoc.2021.675530

**Published:** 2021-05-07

**Authors:** Michal Beno

**Affiliations:** Institute of Technology and Business in Ceske Budejovice, Ceske Budejovice, Czechia

**Keywords:** E-working expenditure, affordability of home, commuting, cappucino, coffee

## Abstract

The aim of this research study is to contribute to the sustainability of e-working initiatives, specifically by exploring three employee-financial impacts. The study adopted a two-step methodological approach: firstly, a comprehensive review of the existing literature was done and, secondly, secondary data analysis was carried out. The study analyses how three potential financial benefits for e-workers differ in various countries and whether these can increase e-workers’ earnings. It was found that there are significant benefits. Regarding the affordability of a home, e-working presents a useful tool to move to less costly regions. This reduces the struggle with housing. Aside from housing, commuting is one of the largest time and cost consuming expenses. Based on our calculations, all employees can save commuting time and money by using public transport or their own vehicles. Important considerations here are the costs, such as fuel, insurance, tolls, time, health and environment. Making cappuccino or coffee at home is a useful way to decrease expenses compared to buying them at a café. Generally, the results indicate that an increase of e-working tends to decrease selected expenses of employees. This paper point outs that, on the basis of average costings, e-working decreases selected expenses of employees. The findings also indicate that more long-term and comprehensive studies are needed, especially in relation to other benefits, such as lunch, childcare and clothing. This study has contributed to highlighting the e-working financial benefits for e-workers by not commuting to work.

## Introduction

E-working (teleworking, telecommuting), in brief, working from home, is not a new concept. The ILO has estimated that 7.9% of the global workforce (5.6% men and 11.5% women) work from home (this includes face-to-display workers) on a permanent basis (ILO, 2021). With the Covid-19 pandemic, the number of employees working from home has increased dramatically, especially in the white-collar workers’ sector (Belzunegui-Eraso and Erro-Garcés, 2020), but people with the lowest incomes and educational attainment have been disproportionately affected (Lund et al., 2020). Recent results of a survey from *Argentina* show that 80% of the workforce was engaging in telework as a policy of social isolation (Sanchéz Zinny, 2020).

Since the pandemic outbreak, there have been some attempts to determine the teleworkability potential. Sostero et al. (2020) estimate that 37% of dependent employment in the EU is currently teleworkable. Dingel and Neiman (2020) found that in the United States the same percentage of jobs is performed entirely at home. Additionally, Saltiel’s (2020) results show that only 13% of workers in developing countries could work from home, yet this share ranges from 5.5% in Ghana to 23% in Yunnan (China). In Australia, almost 40% of all jobs can be done from home (Ulubasoglu and Onder, 2020).

In the literature, a great deal of attention is paid to the potential benefits of e-working from the employee’s perspective (Bernardino and Ben-Akiva, 1996; Mokhtarian and Salomon, 1997; Mokhtarian et al., 1998; Kitou and Horvath, 2003; Shafizadeh et al., 2007).

The object of this research is to contribute to the sustainability of e-working initiatives, specifically by exploring the employee-financial impacts. The impact of the costs for employees varies. Employees can save considerably by e-commuting but, other costs for employees, so-called direct costs (fuel, electricity and air-condition use, mobile phones), increase. The impact of e-working is not equally distributed among different types of employees, employers and countries. To achieve the object of this research, we set out to understand this impact by analyzing secondary data of variables, namely housing, commuting and cappuccino/coffee. The affordability of housing has long been a global problem (Makridis, 2019; Eurostat, 2021). Secondly, commuting has also been tied to increases in labor costs and losses in productivity (Grinza and Rycx, 2020). Finally, coffee is a typical employee habit during an ordinary working day (Coffee and Health, 2017) and increases the expenses (So Pure, 2019) of different generations, genders and industry sectors (PBFY, 2019). This analysis will show the increased disparity between e-workers and intensive face-to-face workers who are generally paid less.

The main research questions in this study were:

• to analyze how three potential financial benefits for e-workers differ in various countries

and

• whether these can increase e-workers’ earnings.

The next section presents a literature review. In the second part, the methodology is introduced. The results of the study are presented in the following section. The fourth section is the discussion, and the last part concludes with a summary.

## Theoretical Background

In the pre-Covid 19 period, European countries were reluctant to implement e-working (Beno and Hvorecky, 2021). But e-working has been a growing phenomenon in the advanced world compared to developing countries (Ansong and Boateng, 2018). This is revealed in recent studies, where it is shown that nearly 100 million workers in 35 advanced and emerging countries could be at high risk because they are not able to work remotely. The poor, the young and women are most at risk (Brussevich et al., 2020). Dingel and Neiman (2020) indicate that the developed world has a low prevalence of jobs that are compatible with working from home compared to the advanced world. Saltiel (2020) estimated that only 13% of employees from ten developing countries could work from home. Delaporte and Pena (2020) evaluated the share of individuals working from home from 7% in Gautemala to 16% in the Bahamas. This is caused by occupational structures [higher-skilled vs. lower-skilled jobs (Watson, 2020)], age, gender and education (Brussevich et al., 2020), and income level (ILO, 2020).

Telecommuting, virtual office and telework are a few of the terms used to describe the same phenomenon (Siha and Monroe, 2006). Simply, e-workers are those workers who are working outside the organisation’s premises using modern technology. Beňo and Ferenčíková, (2019) believe that e-working is a triple-win option.

Working from home has various benefits. According to data from Citrix (2019), potential United States economic gains from a flexible working culture could amount to approximately $2.36 trillion per annum. Possible employee benefits can be brought about by employers encouraging their employees to do telework (Deloitte, 2011). E-working can reduce costs for both employer and employee (Madsen, 2003). But Bernardino (1996) suggested that the expected perceived savings and the actual savings may differ. In this study, the financial benefits have been examined.

According to Riswadkar and Riswadkar (2009), employees can derive a range of cost savings through teleworking. Lister and Harnish (2011) report that e-working saves employees time and money. These authors also say that telework could save employees between $250 and $2700 a year (Lister and Harnish, 2013).

Some of the employees’ work related expenses decrease (Kanellopoulos, 2011), e.g., travel or professional work outfits (Riswadkar and Riswadkar, 2009), office clothing and lunches (Ford and Butts, 1991). Another study showed that over half of the participants (57.1%) indicated somewhat of a reduction in monthly expenses (Baard and Thomas, 2010). Saving money is possible by dispensing with the commute to the office (Wienclaw, 2020, *p*. 2). Additionaly, savings are derived from a reduction in fuel and wear and tear of the vehicle (Ford and Butts, 1991) or costs for parking and other transport (Wienclaw, 2020). This confirms the ILO paper (ILO, 2016) that teleworkers can reduce all these costs, including insurance. According to data collected by Garg and van der Rijst (2015), employees could save an average of 822.06 rands per month if they do not travel to work. Dell employees who on average work remotely 10 days a month save about $350 a year in commuting costs (Sahadi, 2016). Furthermore, working remotely on average 2.4 days per working week would amount to cost savings of $44.4 billion on commuting spent on tickets or fuel (Citrix, 2019). Swink (2008), *p*. 862 also adds that homeworking employees may enjoy reduced expenses for work attire, less stress and reduced transportation expenses. Another financial benefit, especially for the sandwich generation (middle-aged adults who are caring for their elderly parents and their own children), is the decreased cost for babysitting/kindergartens/nurses (Lupu, 2017) and after-school activities (Wienclaw, 2020).

## Methodology

In this paper, a two-step methodological approach has been used. Firstly, a comprehensive review of the available existing literature on the financial benefits of e-working is done because a substantive and thorough literature review is the basis for any good research project (Boote and Beile, 2005).

Next, a secondary data analysis was implemented to create a starting point for future analysis. In this study, secondary sources were used to answer the research question because they offer a good collection of existing data. Furthermore, since the material being investigated comprises a large studying unit, it is impractical to study it directly. Sources of secondary data included journals, newspapers, websites and government and other organization records. This includes the relationship between variables and the cause of a particular variable in relation to e-working. Following the exigencies of settled research questions, inquiries were made about appropriate existing archival sources. The research questions were “the dictatorship of the problem” (Vogt, 2008).

On the basis of research questions, we identified appropriate secondary data in these steps: identification, selection, examination, conducting analyses and interpreting results. In contrast to primary data (individual/team of researchers designs, collects and analyses data), analysis of data collected by others was also used. The United States dollar, as the world’s leading currency, was used for the calculations. In order to perform commuting analyses, it is necessary to consider the amount and pattern of travel of the population in the cities surveyed. In London, 27% of workers typically traveled to work by car, 44% by rail, 13% by bus, 9% on foot and 5% by bicycle (GOV.UK, 2019). The 2011 Hong Kong population census shows that there was more mass transport, 70.1% (bus and train), than travel by car and taxi, 10.5% (Census, 2011). Hong Kong has always been less car-dependent than other cities (Cullinane and Cullinane, 2003; Dawda et al., 2019). Moreover, to be efficient, available and productive (Dawda et al., 2019), a commuting system must serve different requirements in the form of multimodal transportation modes, e.g. Kuhnimhof et al. (2006) found that multimodal individuals in Germany tend to use cars several times per week and use public transport less regularly, preferring to commute. Nobis (2007) presented an extensive overview of multimodality (car/bike, car/public transport, bike/public transport, car/bike/public transport). Heinen and Bohte (2014) point to public transport (rail or underground) and cycling as resulting multimodal groups. Cycling as a multimodal option is affected by positive weather conditions (Heinen et al., 2011). The City of London has a long tradition of a multimodal integrated system (Dawda et al., 2019).

The utilization of available data to answer research questions has several benefits (less time and resources are required, access is obtained to large and wide-ranging data sets). Some limitations do occur, including a lack of knowledge of the existence of rich data sets and how to obtain and evaluate the contents, insufficient or outdated data, and lack of funds to hire staff to assist with the work (Dunn et al., 2015).

This analysis strategy is geared toward producing actionable findings. Furthermore, these findings also provide information for further research.

## Findings

Recent articles draw the attention to the most costly elements of e-working. For example, remote work will cost business in Japan more than 1.3 trillion yen (Nakafuji, 2020). Munk (2020) stresses that despite the potential savings (commuting, dry-cleaning bills, shopping for work attire), there are some hidden expenses, such as electricity bills, phone data plans, supplies for the home office and wear and tear of personal devices. Interestingly, a recent CreditCards.com survey shows that working from home due to the pandemic costs households about $108 more per month (Segal, 2020).

### Housing

The main benefit of e-working is that one works from home or another location instead of working in a cubicle. A recent study by Redfin revealed that a quarter of participants move for reasons of affordability, and almost 60% said their ability to afford non-housing expenses and leisure activities improved after their relocation (Katz, 2020).The latest data reveal that 14 million to 23 million Americans are planning to benefit from e-working by seeking housing in more affordable markets, with the biggest out-migration occurring in major cities (Upwork, 2020).

Generally, people choose where to live on the basis of their workplace and try to avoid long commutes. But times have changed due to the high housing prices around the world. Based on a Demographia International Housing Affordability Survey, the 10 least affordable international housing markets ranked by house price to income ratio were Hong Kong, Vancouver, Sydney, Melbourne, Los Angeles, Toronto, Auckland, San Jose, San Francisco and London (Cox and Pavletich, 2020). Notably, New Zealand, Hong Kong, the United States, the United Kingdom and Australia are the top regions doing business (WBG, 2020), but they are at the top of those with the least affordable housing. Through e-working, doing business remotely offers employees the opportunity to participate/work without being present. Conversely, baby boomers, on account of their age and children, were considering downsizing and moving back into cities where walkability and urban living are more appealing. E-working seems to be the catalyst (Schreiber, 2016). This is in accordance with the life course rather than economic motives in residential relocation decisions (Nijkamp et al., 1993). As technology continues to improve and these housing trends increase, more employees will recognize the benefits of working remotely. The empirical evidence indicates that using the home as a workplace has become a substantial feature of labor markets in many western economies and that this has increased significantly in recent years (Doling and Arundel, 2020).

### Commuting

One of the benefits of e-working is the decrease of traffic congestion and lower carbon emissions (Kitou and Horvath, 2003; Shafizadeh et al., 2007). But transport costs have a significant impact on the structure of economic activities as well as on international trade. Empirical evidence underlines that raising transport costs by 10% reduces trade volumes by more than 20% (Rodrigue and Notteboom, 2020).

Commuting is seen as one of the largest time-consuming (traffic or waiting for delayed public transport modes) and recurrig expenses (fuel, parking, tolls, montly transit passes) that mankind faces. One study presented the best and worst commute cities among 74 worldwide cities (Expert Market, 2019). In Table 1, cost-saving calculations show that London‘s monthly ticket public transport is the most expensive one, and also show that besides long commutes and barriers, cost savings could be the reason for further e-working expansion. Researchers found that an extra minute of commuting time reduces both job and leisure-time satisfaction, but increases strain and worsens mental health for workers. Additionally, 20 min of commuting to and from work each working day have the same effect on job satisfaction as a 19% reduction in personal income (UWE, 2017). Recently it was found that Londoners commute 74 min each day (NE, 2020) and spend $179.40 each month for a ticket pass (Deutsche Bank, 2019).

**TABLE 1:** Commuting (London).

Commuting days	Tickets per week	Monthly ticket public transport	Monthly savings	Annual savings
5	10	$179.40	$0	$0
4	8	$143.52	$35.88	$430.56
3	6	$107.64	**$71.76**	**$861.12**
2	4	$71.76	$107.64	$1291.68
1	2	$35.88	$143.52	$1722.24
0	0	$0	$179.40	**$2152.80**

Source, Author’s own calculation based on Deutsche Bank, 2019.

Based on this calculation, Londoners who choose hybrid working (partly at home and partly in cubicles) at least 2 days could save $71.76 monthly and $861.12 annually, compared to $2152.80 annually by working remotely full-time, an almost threefold difference (see highlighted data in Table 1).

By commuting, drivers incur a number of costs. In Table 2, calculations of overall savings for Hong Kong are presented.

**TABLE 2:** Commuting by car (Hong Kong).

Commuting days	Average distance km^a^	Monthly savings spent on fuel^b^ ^,^ ^c^	Monthly savings spent on parking^d^
5	308.40	$0	$0
4	246.72	$147.91	$138.44
3	185.04	$295.82	$276.88
2	123.36	$443.73	$415.32
1	61.68	$591.93	$553.76
0	0	**$739.54**	**$692.20**

a Average distance 15.42 km (Numbeo, 2021).

b 20 working days.

c Average cost per gallon of fuel $2.40 (GPP, 2021).

d Daily parking cost $34.61 (Spacer, 2021).

Source, Author’s own calculation.

Commuters spend on average 42.42 min (Numbeo, 2021) a day commuting - 14 h a month or 170 h per year - which is almost comparable to the United States rate (Vasel, 2015). The average Hong Kong commuter driver spends over $739.54 monthly on fuel and $692.20 monthly (as highlighted in Table 2) on daily parking (28% of men’s monthly income and 37% of female’s monthly income (Stotz, 2021)), or $352 on a monthly basis (Spacer, 2021) on parking alone (14% of men’s monthly income and 19% of females’ monthly income (Stotz, 2021)), which could be saved in its entirety by e-working, but only 3.12% of employees in Hong Kong work from home (Numbeo, 2021). Saving money on cost components such as fuel, insurance, tolls, time, health and the environment should be further considered.

### Coffee

Coffee is celebratory. Its consumption is uncommon, and its uncommonness imbues it with a unique mystique associated with a wealthy, refined and intellectually evolved class. Coffee is predominantly an outside drink, it derives its utility from a social, esthetic and emotional role (Verma, 2013). Coffee has become an important part of societal norms across the globe (Waxman, 2004; Almqvist et al., 2007; Moretti, 2017).

Millennials spend $2008 a year at coffee shops (Mckenzie, 2020), and the average American spends about $1100 a year on coffee (Acorns, 2020). Furthermore, the data highlight that 41% of them admitted to spending more on coffee in the past year than they had invested in their retirement savings (Mckenzie, 2020).

Generally, we save money by making things to eat or to drink at home (Thorpe, 2012). McClean et al. (2020) found that something as simple as missing one’s regular morning cup of coffee can cause that employee to begin the workday less calm and more mentally exhausted. The costs for coffee or cappuccino vary from country to country and from continent to continent (CCM, 2019; Deutsche Bank, 2019). A cappuccino in Copenhagen costs $6.30 (Deutsche Bank, 2019), and a coffee costs $6.24 in Doha (CCM, 2019). We have calculated that making morning cappuccino at home costs $0.50 and coffee costs $0.20. As shown in Table 3, making morning cappuccino and coffee at home is cheaper than buying it. An employee in Copenhagen who drinks one morning cappuccino on 5 days a week spends $126 per month, but when e-working full-time he/she saves $116 monthly and $1392 annually. Furthermore, when drinking one morning coffee an employee spend $124.80 per month, but when e-working full-time he/she saves $120.80 monthly and $1449.60 annually. Making one’s own cappuccino or coffee is clearly a way to save money with working remotely, whether it is full-time or not.

**TABLE 3:** Morning cappuccino and coffee.

Commuting days	Monthly cappuccino at a cafe	Monthly cappuccino at home	Monthly coffee at a cafe	Monthly coffee at home
5	**$126.00**	$0	**$124.80**	$0
4	$100.80	$2.00	$99.84	$0.80
3	$75.60	$4.00	$74.88	$1.60
2	$50.40	$6.00	$49.92	$2.40
1	$25.20	$8.00	$24.96	$3.20
0	$0	$10.00	$0	$4.00

Source, Author’s own calculation.

## Discussion

E-working can help to reduce the employee’s expenses, as shown in this study. Is it possible for e-working to affect housing choices? The rise of e-working (developing economies adapt more easily (Bana et al., 2020)) enables employees to live and work away from the organisation’s premises, even a long way away. The data in this study confirm that the shift to working remotely has changed and will continue to change housing patterns. A world in which e-workers work at home may not only affect the form and choices of housing, but also implies a potential transformation of the physical structure of cities, the distribution of property values and the broader labor and housing markets themselves (Doling and Arundel, 2020). While the pandemic may have made offices less accessible, hybrid working, co-working and e-working will be much appreciated in the future. Wilsker (2008) states that the average full-time e-worker, regardless of salary scale, receives the equivalent of an annual salary increase of $8400 due to the reduced expenses that result from e-working (vehicle, clothing, parking, food and insurance costs). Some of these expenses were examined in this study with positive potential savings for employees. What does it mean in the long term? As also shown in this study, some trends are already emerging. E-workers are more likely than commuters to reside in more peripheral areas, such as urban green settings or town and rural areas, as stated in an analysis from the Netherlands (Muhammad et al., 2007), where the rate of e-working is very high.

## Conclusion

In the pre-Covid era, very few employees worked from home (and this was usually on a casual basis). Suddenly, e-working exploded even in developing countries. To work from home situation is expected to continue. The impact of e-working is not equal divided around the globe among different types of employees including age, gender, education, skills and occupational structures.

In this study, we discussed and analyzed three financial benefits of e-working. Three dependent variables were used: housing, commuting and cappuccino/coffee. The study was conducted in two stages. The first stage investigated the financial benefits for e-working. In the second stage, dependent variables were analyzed.

The main research question investigated in this paper is:

*How three potential financial benefits for e-workers differ in various countries and whether these can increase e-workers’ earnings?* Our analysis considers both quantifiable and non-quantifiable considerations. It is clear that e-workers can reduce their expenses compared to regular commuters, even in hybrid or full-time work from home. We set out to research how, in terms of costs and benefits (housing, commuting and cappuccino/coffee) a permanent increase in working from home would influence overall employee expenses. It was found that increasing the period of working from home is driven by cost savings for employees. This translates to a reduction of the struggle for housing, less commuting and saving money by drinking a cappuccino/coffee at home. However, in those countries with a greater share of informal economy and a low prevalence of remote jobs, the result can be the reverse.

As modern technology continues to improve, housing trends will accelerate, commuting will become more expensive and time consuming, and prices will increase. More employees are likely to recognize the savings potential that comes from working from home instead of commuting to work. Offering a flexible work culture including e-working can be a profitable long-term strategy. If costs continue to be lower than those for on-location, more employers will increase at-home work. The latest data of one study indicate that when workers experience higher WFH efficiency, they have a higher preference for WFH even after the pandemic. Additionally, female workers preferred WFH twice per week, while the male workers more often preferred WFH once per week. Finally, workers from the management and the self-employed levels demonstrated a lower preference for WFH, compared to the front-line and middle-grade workers (Wong et al., 2020). Obviously, however, some jobs still have to be carried out on site.

The limitations of the study arise mainly from not taking into consideration mitigating, location-cultural specific expenses in relation to dependent variables explored in this study (e.g., air conditioning, electricity, free snacks or lunches). Moreover, the modality of groups described in the methodology give rise to further limitations. Nobis (2007) notes that most adolescents are multimodal, and Kuhnimhof et al. (2006, 2012) determined that single people are multimodal.

## Further Research

The labor market impact of Covid-19 still need to be examined. Aspects not covered by this research are the analysis of lunch, childcare and clothing variables. These issues could be dealt with in future research. This is needed in order to understand how additional data can throw further light on e-working as a beneficial option. But companies and organisations that allow their employees to work remotely can reap benefits too. More research should be done on the benefits for employers in the future.

## Data Availability

The original contributions presented in the study are included in the article/Supplementary Material, further inquiries can be directed to the corresponding author.
